# Isolation, characterization and complete genome analysis of a novel bacteriophage vB_EfaS-SRH2 against *Enterococcus faecalis* isolated from periodontitis patients

**DOI:** 10.1038/s41598-022-16939-0

**Published:** 2022-08-02

**Authors:** Setareh Pazhouhnia, Majid Bouzari, Farahnaz Arbabzadeh-Zavareh

**Affiliations:** 1 Department of Cell and Molecular Biology and Microbiology, Faculty of Biological Science and Technology, University of Isfahan, Hezar-Jereeb Street, Isfahan, 81746-73441 Iran; 2 Department of Operative Dentistry and Torabinejad Dental Research Center, School of Dentistry, Isfahan University of Medical Sciences, Isfahan, Iran

**Keywords:** Sequencing, Microbiology, Bacteriophages, Virology

## Abstract

Periodontitis is a chronic inflammatory condition that can damage soft tissues and supporting teeth. *Enterococcus faecalis* is an opportunistic pathogen usually living in the oral cavity and plays a critical role in apical periodontitis that significantly threatens human health. The use of bacteriophages as an alternative way to eliminate bacterial infections is a promising approach. *E. faecalis* was isolated from the depth of dental packets of patients with periodontitis. Antimicrobial susceptibility was tested using 16 antimicrobial agents. Also, a specific virulent bacteriophage (vB_EfaS-SRH2) with an irregular pentagonal morphology of the head and a non-contractile tail belonging to the *Siphoviridae,* was isolated from wastewater in East of Isfahan, Iran, and its physiological and genomic specifications were investigated. The genome was double-strand DNA with 38,746 bp length and encoded 62 putative ORFs. In addition, eight Anti-CRISPERs and 30 Rho-dependent terminators were found. No tRNA was found. It had a short latent period of 15 min and a large burst size of _~_ 125. No undesirable genes (antibiotic resistance, lysogenic dependence, and virulence factors) were identified in the genome. Based on physiological properties and genomic characteristics, this phage can be used as a suitable choice in phage therapy for periodontitis and root canal infection.

## Introduction

Periodontitis is commonly known as gum disease and damages both soft and hard tissues covering the tooth root^1^. Overuse and abuse of antibiotics have led to increasing pathogenic bacterial strains resistant to antibiotics^2^. *Enterococcus faecalis*, along with other microorganisms, are the causative agents of periodontitis and tooth root infections^3^. It often causes infections in the tooth root canal and asymptomatic root treatment resistance. The elimination of *E. faecalis* from the root canal is difficult due to the formation of biofilm and resistance to antibiotics. It can also escape the host immune system due to the hiding in the root canal^4^. Although this bacterium is rare in endodontic infections, it can replicate in the filled root canal and spread outside the root and finally causing apical periodontitis^5^. The mechanical elimination of microorganisms in periodontal pockets by surgery and non-invasive techniques is time-consuming, costly, wearying, and some fail to inactivate pathogens beneath the gum^6^. Therefore, this necessitates implementing alternative methods. Using bacteriophages against pathogenic bacteria is one of the promising essential treatments. Compared to chemotherapy, phage therapy has many advantages. They do not damage the natural flora and do not have any side effects^7^. Lee et al. studied vB-EfaS-HEf13 phage of the family *Siphoviridae* isolated from the oral cavity against *E. faecalis*. They evaluated the lytic activity of the phage against *E. faecalis* in sections of the dentin under the scanning electron microscope (SEM). They concluded it could be used as a suitable therapeutic agent against refractory apical periodontitis associated with *E. faecalis*^8^. Bachrach et al. screened the saliva samples from 31 donators for the presence of bacteriophages and reported phages acting against *E. faecalis* in seven cases^9^. Recent evidence shows that phages may often involve in preventing periodontal infections because of phage-based components discovered in the gum sections in moderate to severe periodontitis^10^. Considering the resistance of *E. faecalis* against drugs prescribed, phage therapy is proposed as a new approach to treat oral cavity complications^9^.

This study aimed to isolate and phenotypically and genetically identify *E. faecalis* strains from people with periodontitis and also, isolation and characterization of a novel virulent phage against *E. faecalis* for possible phage therapy of periodontitis and root canal infection.

## Results

### *E. faecalis* isolation and identification by phenotypic and genotypic characteristics

**Figure 1:**
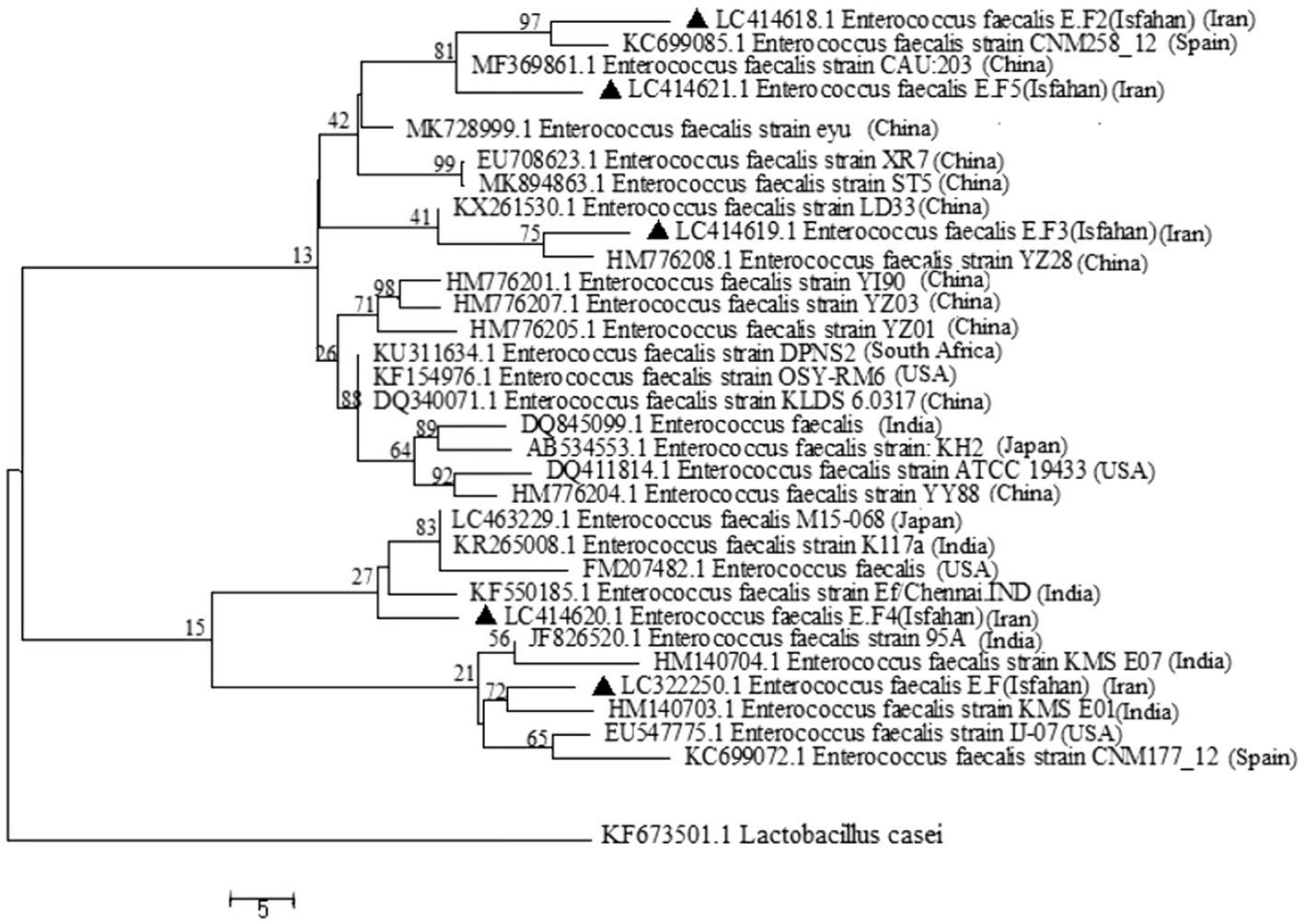
Phylogenetic tree of the *E. faecalis* isolates based on 16S rRNA sequences. The maximum likelihood tree was built using *Lactobacillus casei* (KF673501.1) as an out-group with 1000 bootstrap replicates using MEGA 7 software. Branch numbers show bootstrap percentage following 1000 replications. Scale bar corresponds to phylogenetic distance of 0.05 nucleotide change or substitutions per site. ▲: Indicates *E. faecalis* isolated in this study.

Among the colonies observed on Mitis Salivarius Agar, dark blue colonies were Gram stained. According to the observation of Gram-positive cocci with individual, paired or short chains arrangements and in the biochemical test being negative in the catalase, tolerating 6.5% salt, growth at 10 and 45 °C, and hydrolysis of esculin in the presence of 40% bile, 13 out of the 206 collected samples were diagnosed as *E. faecalis.* In PCR confirmatory test, expected bands of ~ 370-bp were observed (Supplementary Fig. S1). Some of the 16S rRNA gene sequences were deposited in Genebank with accession numbers of LC322250.1, LC414618.1, LC414619.1, LC414620.1, and LC414621.1. In the phylogenic tree constructed (Fig. 1), *E. faecalis* isolates were diverse and distant and placed in different clusters being close to the species reported from China, Japan, and the USA.

### Antimicrobial susceptibility

**Figure 2:**
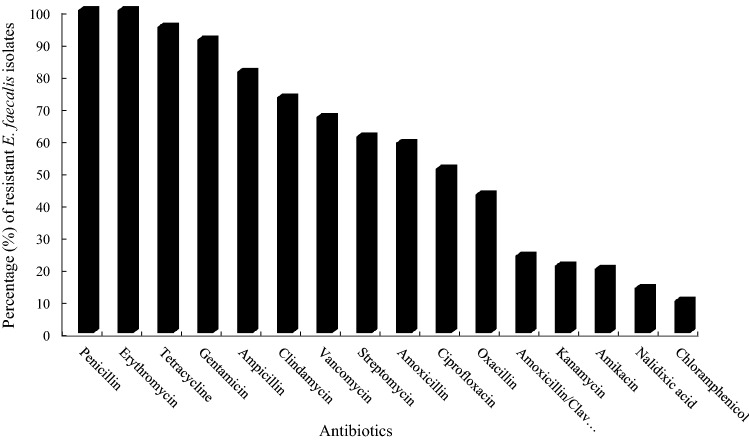
Percentage of antimicrobial resistance of fifteen *E. faecalis* strains to different antibiotics. Penicillin (PEN), Erythromycin (ERY), Tetracycline (TET), Gentamicin (GEN), Ampicillin (AMP), Clindamycin (CLI), Vancomycin (VAN), Streptomycin (STP), Amoxicillin (AMX), Ciprofloxacin (CIP), Oxacillin (OXA), Amoxicillin/Clavulanic acid (AMC), Kanamycin (KAN), Amikacin (AMK), Nalidixic acid (NAL), and Chloramphenicol (CHL).

Profiles of *E. faecalis* resistance to antimicrobial agents are shown in Fig. 2. All the isolates were resistant to penicillin and erythromycin (100%), while the lowest resistance rate was observed for chloramphenicol (10%) and nalidixic acid (14%). Resistance rate for tetracycline, gentamycin, ampicillin, clindamycin, vancomycin, streptomycin, amoxicillin, ciprofloxacin, oxacillin, amoxicillin/clavulanic acid, kanamycin, and amikacin were 95%, 91%, 81%, 73%, 67%, 61%, 59%, 51%, 43%, 24%, 21% and 20%, respectively.

### Morphology of the phage

**Figure 3:**
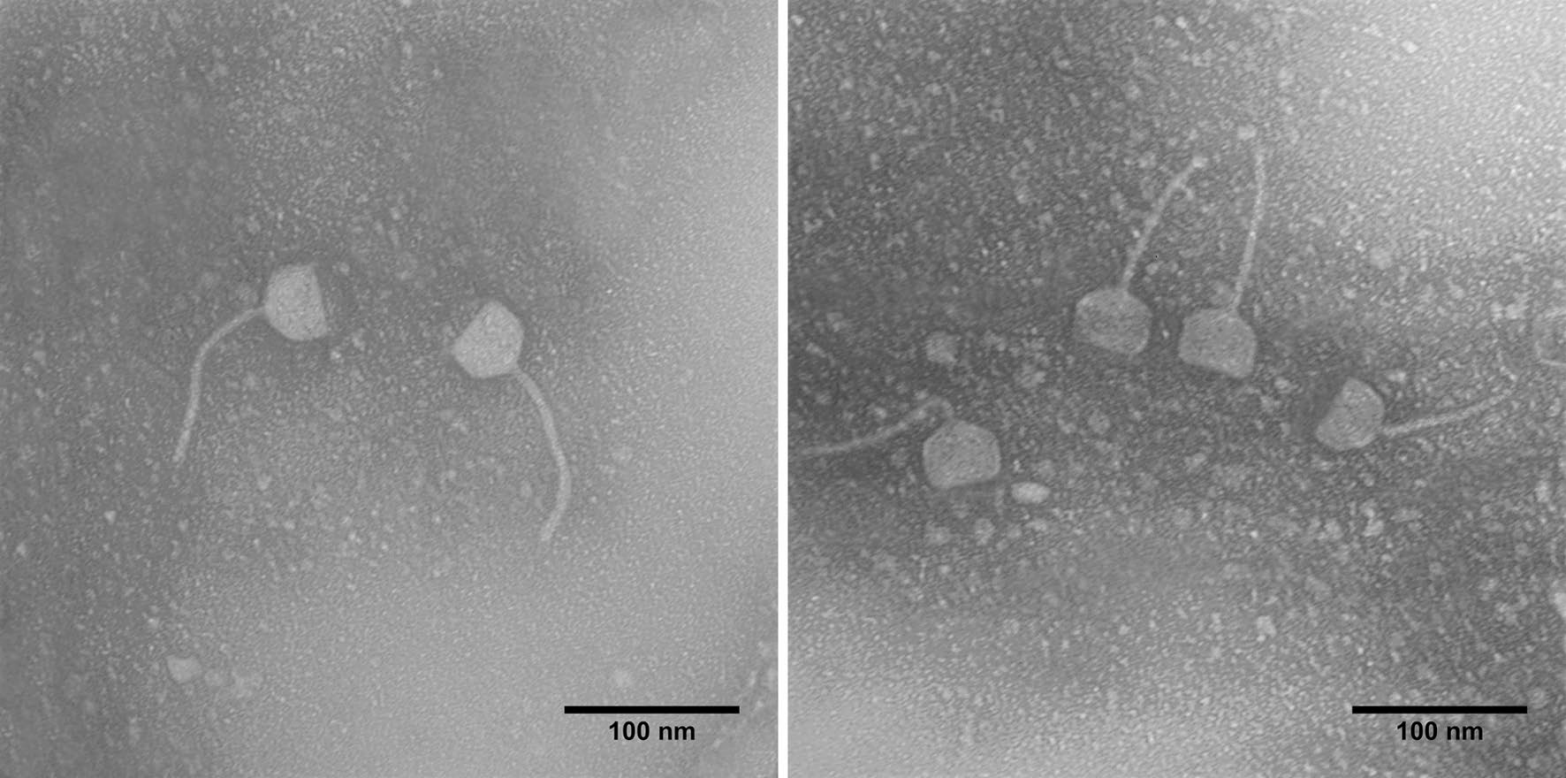
Electron micrograph of the vB_EfaS-SRH2 phage with pentagonal heads and flexible tails.

A virulent phage was isolated from a wastewater treatment plant, East of Isfahan City. Clear and bright plaques were observed with ~ 1–2 mm diameter (Supplementary Fig. S2). It was named vB_EfaS-SRH2 according to Kropinski et al.^11^. In electron microscopy, a unique pentagon head with 42 ± 1 × 34 ± 1 nm in diameter and a flexuous and non-contractile tail (103 ± 1 nm length and 6 ± 1 nm width) were observed. Concerning morphology, the obtained phage was diagnosed as a member of the *Siphoviridae* family (Fig. 3).

### Phage host range

Among all various standard strains, only *E. faecalis* ATCC 29212 and clinical isolates of *E. faecalis* were sensitive to it. The EOP of the vB_EfaS-SRH2 phage on the isolated *E. faecalis* and *E. faecalis* (ATCC 29212) were investigated. EOP was classified into three levels high production (≥ 0.5), medium production (0.2–0.5) and low production (0.2–0.001). The EOP of 61.53% of the isolates wa < 0.5, while for 23.07% and 15.38% of the isolates it was 0.2–0.5 and 0.2–0.001 respectively (Supplementary Fig. S3).

### The effects of thermal, pH, and saline stress on the phage

**Figure 4:**
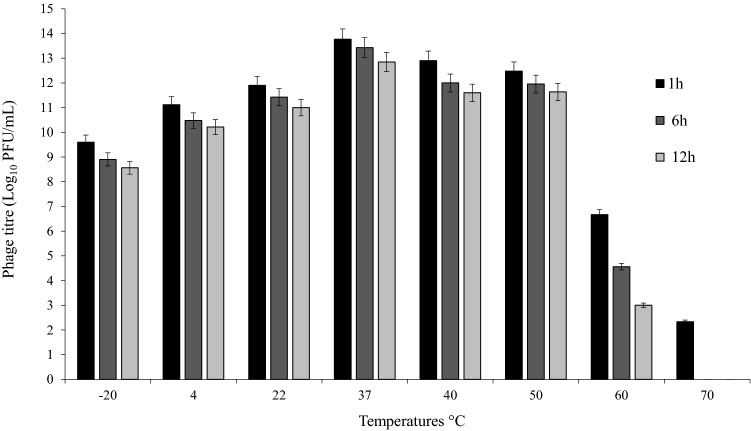
Stability of vB_EfaS-SRH2 phage at different temperatures (− 20 to 70 °C). The results are presented as the mean ± SD. Error bars indicate the standard deviation.

**Figure 5:**
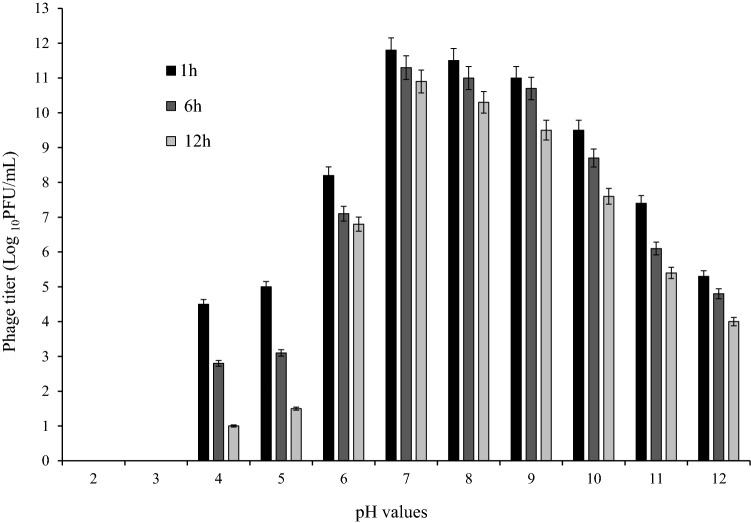
Stability of vB_EfaS-SRH2 phage at different pH values (2–12). The results are presented as the mean ± SD. Error bars indicate the standard deviation.

**Figure 6:**
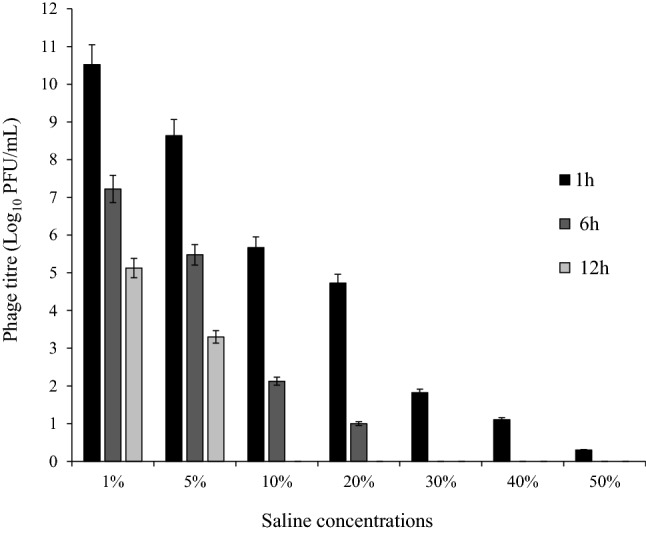
Stability of vB_EfaS-SRH2 phage at different saline concentrations (1–50%). The results are presented as the mean ± SD. Error bars indicate the standard deviation.

The effects of thermal, pH, and saline stresses on the phage are shown in Figs. 4, 5, 6. The phage had high activity in 1, 6, and 12 h at − 20 to 50 °C, with maximum activity at 37 °C. The titer of the phage was significantly declined at 70 °C after 6 h (P < 0.05) (Fig. 4). Maximum phage activity was observed at pH 7. Although the phage was able to be active in a wide pH range, its activity was better in alkaline pH (7–12). It was inactivated entirely at pH 2 and 3 (Fig. 5). Maximum infection activity was observed for 1% saline concentration within 1 h. Phage activity decreased significantly from 30% saline concentration and above (P < 0.05) (Fig. 6).

### Phage adsorption rate, burst size, and latent period

**Figure 7:**
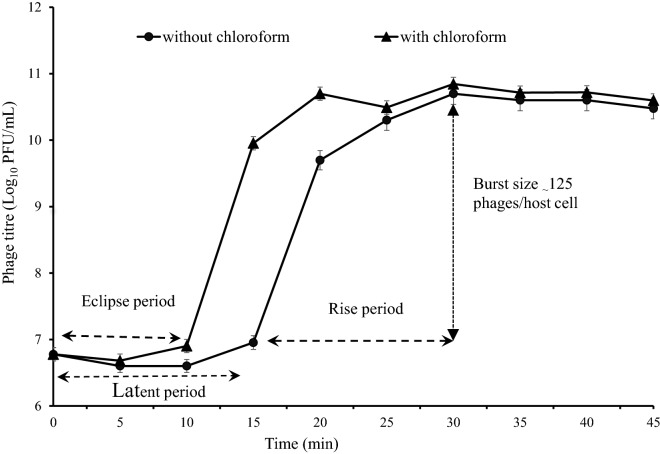
Curve for one-step growth of the vB_EfaS-SRH2 phage, showing latent, rise and eclipse periods, and also burst size. Closed circles indicate non-chloroform-treated samples; closed triangles indicate chloroform-treated samples. Data are presented as mean ± SD from three independent experiments. Error bars indicate standard deviations.

The one-step growth curve analysis showed that the latent and rise periods of this phage both were 15 min and the burst size was _~_ 125 phages per host cell (Fig. 7). In addition, 88% of the phage particles were adsorbed on the *E. faecalis* cell after 10 min (Supplementary Fig. S4).

### Bacterium reduction assay or multiplicity of infection

**Figure 8:**
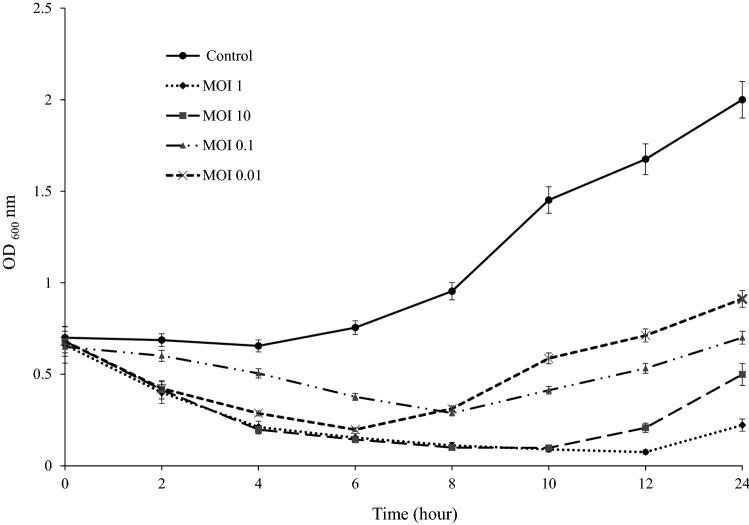
MOIs of vB_EfaS-SRH2 phage for *E. faecalis*. Error bars represent standard deviations from three independent.

**Figure 9:**
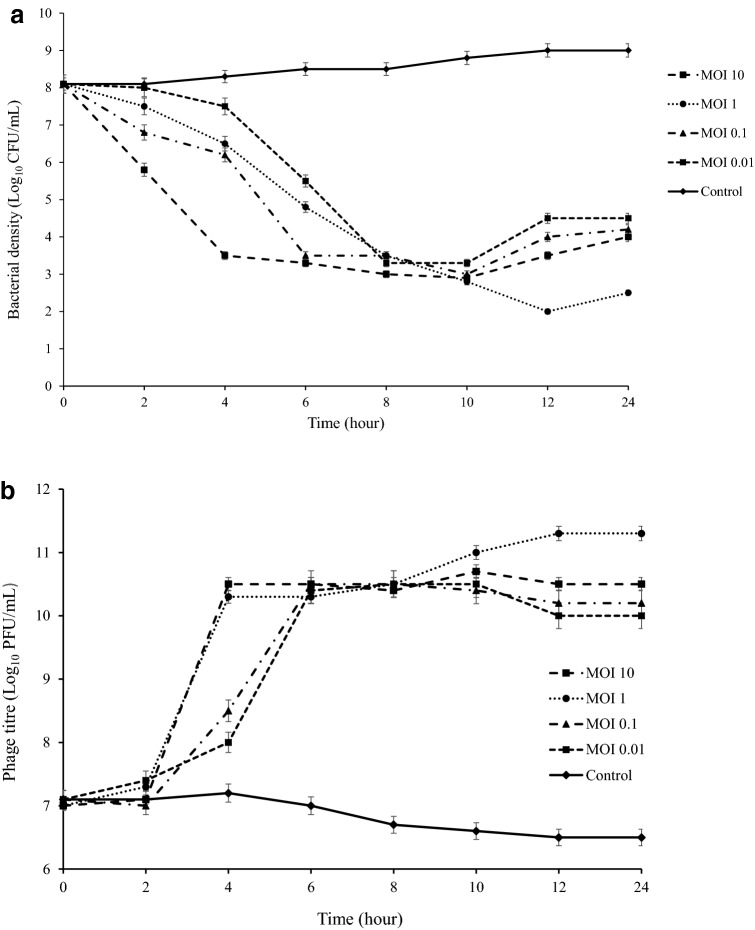
Kinetics of lytic development of the vB_EfaS-SRH2 phage in *E. faecalis*. (**a**) A number of surviving cells after the phage infection per 1 mL (CFU/mL), and (**b**) a number of phages per 1 mL (PFU/mL). The data represent the average of three independent experiments. Error bars represent the standard deviation.

The vB_EfaS-SRH2 phage infection of *E. faecalis* could ultimately decrease *E. faecalis* in all MOIs after 24 h. A sharp decrease in the growth of *E. faecalis* was observed in 4, 6, and 8 h at MOIs 10, 0.1, and 0.01, respectively. The highest activity was observed at MOI 1 after 12 h (Fig. 8). Also, the lysis of *E. faecalis* was fast and complete in the presence of phage (Fig. 9a,b).

### vB_EfaS-SRH2 phage genome sequencing and analysis

**Figure 10:**
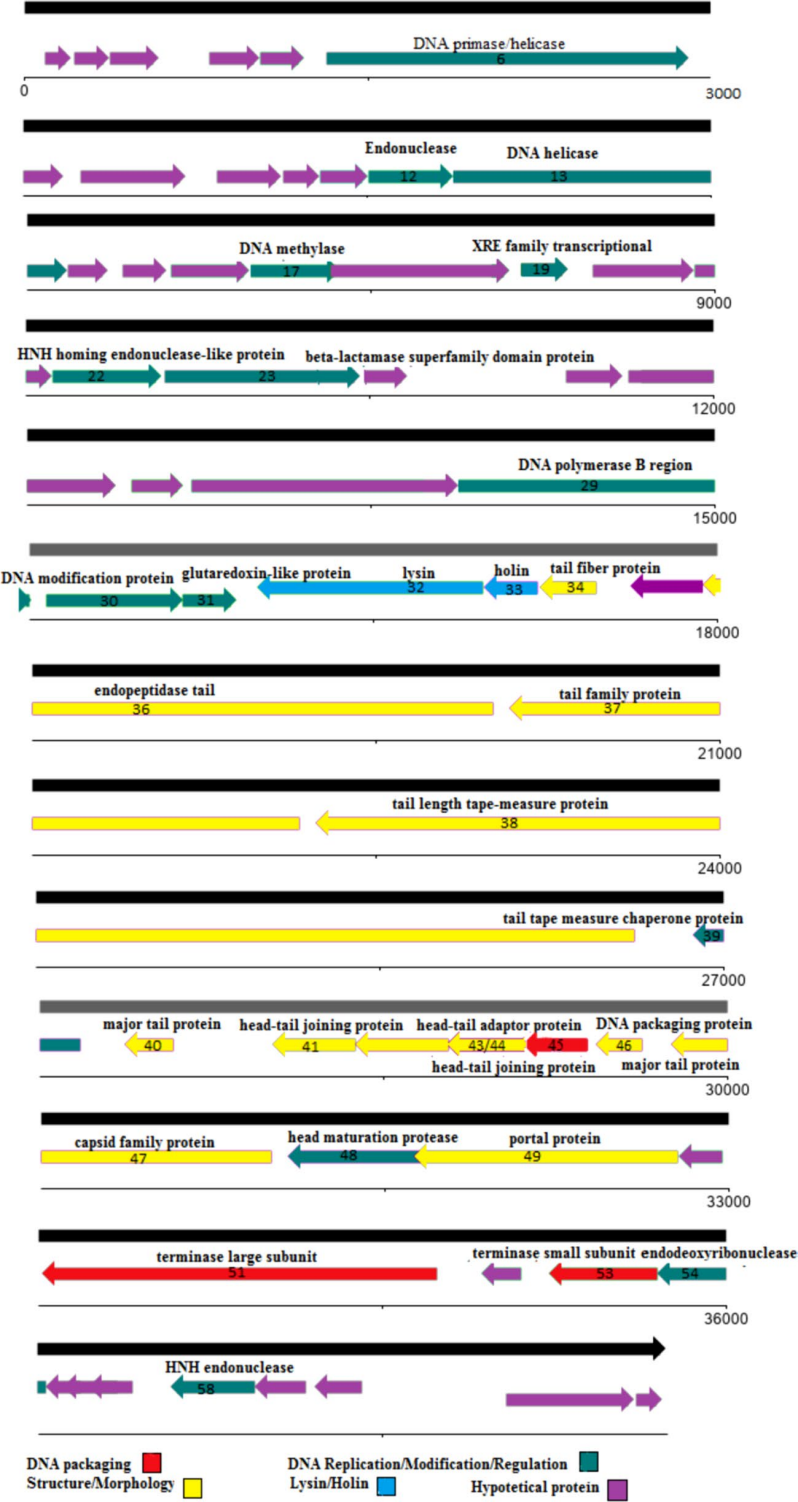
Genetic mapping of the vB_EfaS-SRH2 phage genome using DNA Plotter software. Arrows indicate the predicted 62 ORFs for this genome with their corresponding functions in different colors (red, green, purple, yellow, and blue).

**Figure 11:**
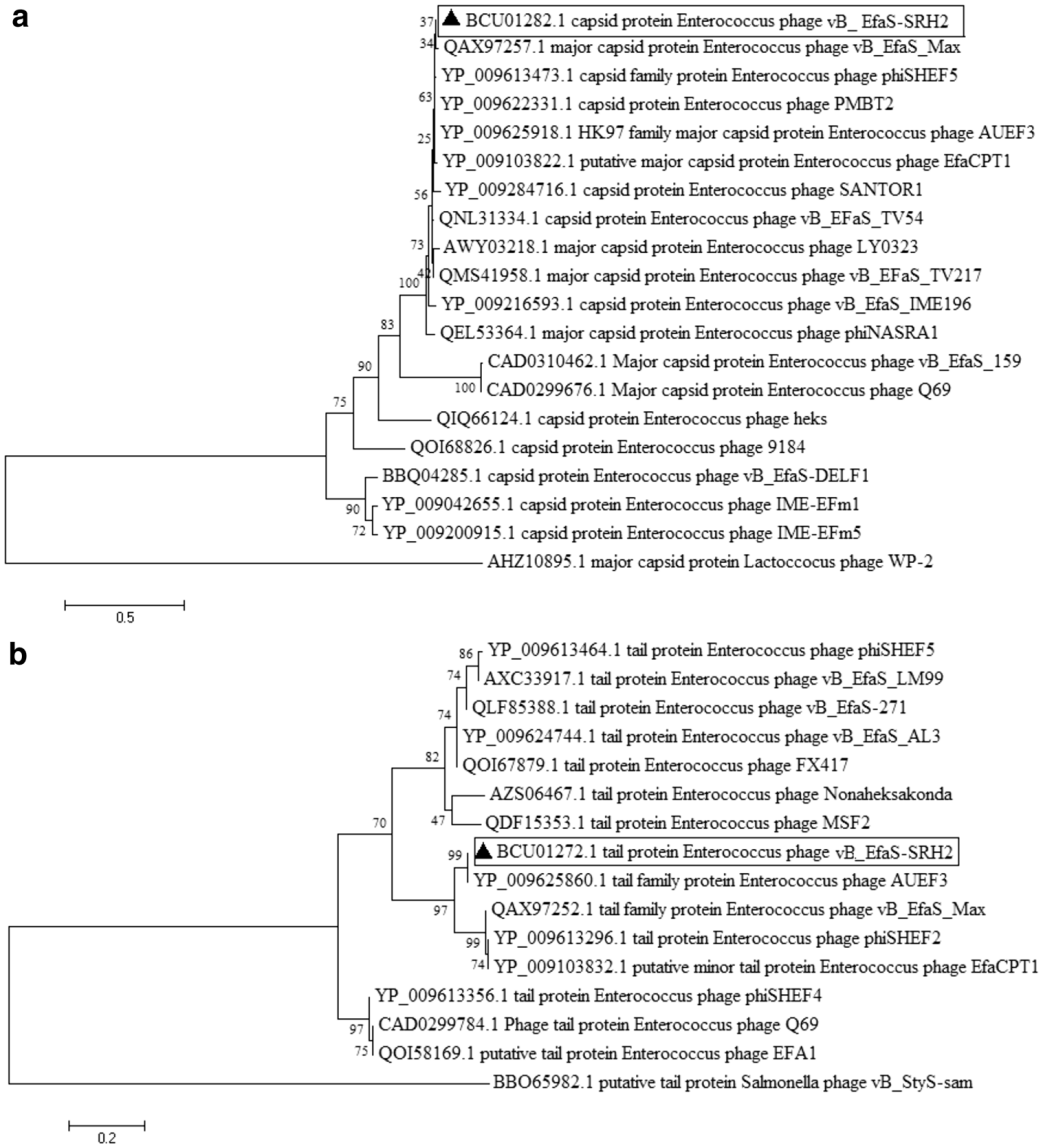
(**a**,**b**) Phylogenetic relationship of vB_EfaS-SRH2 phage. Phylogenetic trees drawn based on amino acid sequences of the capsid protein (**a**) and tail family protein (**b**) with MEGA 7 software with 1000 bootstrap replications. ▲: sequences of the phage detected in this study.

The vB_EfaS-SRH2 phage has double-stranded linear DNA with 38,749 bp length and 34.78% G + C content. It contains 62 ORFs in which 33 (53.22%) and 29 (46.78%) ORFs are placed on positive and negative strands, respectively. Among them, 35 (56.45%), 13 (20.96%), and 14 (22.58%) ORFs end to TAA, TGA, and TAG, respectively (Supplementary Table S1). All the predicted ORFs begin with the ATG codon. The tRNA coding sequence was not found by GtRNAdb and tRNAscan-SE software. Genome analysis by using ARNOld software showed 30 Rho-dependent terminators in the genome structure of this phage. Eight Anti-CRISPERs were found, including ORF7, ORF8, ORF9, ORF10, ORF11, and ORF12 on the positive strand and ORF53 and ORF54 on the negative strand. Fifty percent of the reported ORFs code important clusters, including structure, morphology, replication, and regulation. The remaining fifty percent code hypothetical proteins. Twelve ORFs code the structure/morphogenesis cluster, including tail fiber protein (ORF34), tail family protein (ORF37), major tail protein (ORF40, ORF46), capsid family protein (ORF47), and portal protein (ORF49). Twelve ORFs also code the replication/modification/regulation cluster, including DNA primase/helicase (ORF6), endonuclease (ORF12), DNA methylase (ORF17), and HNH endonuclease (ORF58). Two ORFs, i.e., ORF51 and ORF53, encode DNA packaging-related proteins. ORF32 and ORF33 encoded lysin and holin, respectively. The longest ORF in this phage was ORF38, which encoded tail length tape-measure protein in the structure/morphogenesis cluster (Fig. 10). No virulence, lysogenic dependence, and antibiotic resistance genes were found in the vB_EfaS-SRH2 genome analysis. Compared to other reported phages related to *E. faecalis,* the vB_EfaS-SRH2 phage classification by phylogenetic tree based on capsid protein and tail family protein genes (Fig. 11a,b) showed that this phage is a member of the *Efquatrovirus* genus of the *Siphoviridae* family (Supplementary Fig. 5). The vB_EfaS-SRH2 phage can be traced as a new member of *Siphoviridae *under accession number LC623721.1 at NCBI.

### The effect of bacteriophage on biofilm mass

**Figure 12:**
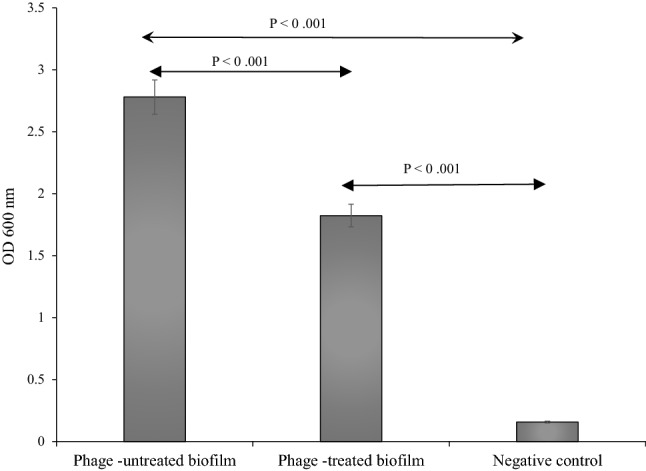
The effect of the phage vB_EfaS-SRH2 on the biofilm mass of *E. faecalis*. The assay was performed in triplicate in wells of polystyrene microtitre plates. Error bars indicate standard deviations. P values < 0.05 were considered significant.

The optical absorptions of bacteriophage-treated biofilm, bacteriophage-untreated biofilm, and negative control were read at OD_600_ nm after 24 h of incubation. Average absorptions for bacteriophage-untreated and treated biofilms were 2.78 (2.64–2.95) and 1.82 (1.65–1.98), respectively. The optical absorption of untreated biofilm was significantly higher than that of the bacteriophage-treated biofilm (P < 0.05), and absorption of both the bacteriophage-treated biofilm and the bacteriophage-untreated biofilm were higher than the culture medium without bacteria (negative control) (P < 0.05) (Fig. 12).

## Discussion

*E. faecalis* is a serious threat to human health, as it is present in many infections, and its high resistance to antibiotics helps its pathogenicity. It is the crucial element in root canal infections and apical periodontitis or resistant apical periodontitis^8,12^. The use of antibiotics is common in dentistry, and this may lead to antibiotic resistance among other oral bacteria and treatment failure^13^. *E. faecalis* isolated from individuals with periodontitis and root canal have a high rate of resistance to tetracycline, erythromycin, clindamycin, and metronidazole^14^. Isolated strains in this study were examined in terms of antimicrobial susceptibility by a wide range of antibiotics and showed resistance to a large number of them. This entails new approaches to cope with antibiotic resistance in the treatment of odontitis and recurrent and untreatable periodontitis. One of these is bacteriophage therapy by virulent bacteriophages^15^. In this study, a phage with a novel morphology was identified. It (vB_EfaS-SRH2 phage) was specific for standard *E. faecalis* and isolated ones and did not affect some other Gram-positive and Gram-negative bacteria tested. The specificity of the phage has a significant effect on achieving the goal of eliminating *E. faecalis*. The sensitivity of various *E. faecalis* strains to the phage was different, and this could be related to the phage protection systems in the host strains.

Most phages are capable of tolerating neutral pH and a wide temperature range. The vB_EfaS-SRH2 phage could tolerate a wide range of pH and temperatures and be tolerant to high temperatures and alkaline pH. These results are consistent with those reported for temperature and pH tolerance of *E. faecalis* phages vB_EfaS_HEf13 (Accession No. MH618488.1), vB_EfaS-DELF1 (Accession No. LC603364.1), and vB_EfaS_PHB08 (Accession No. MK570225.1)^8,16,17^. These characteristics are effective in bacteriophage therapy because the bacterium’s survival at high pHs and temperatures is the characteristic that protects it from antibiotics and neutrophilic phages^4^.

Saline tolerance range is rarely studied in phages reported for *E. faecalis*. The results of tolerance to different saline concentrations of the phage were almost similar to those reported for vB_EfaS-DELF1 and VD13 phages that could not tolerate and function in different saline concentrations^17,18^.

Short latent period, large burst size, and fast adsorption of vB_EfaS-SRH2 phage on the *E. faecalis* surface shows its proper function. It had a shorter latent period and a larger burst size compared to ϕEF24C and IME-EF1 phages^19,20^. Also, the latent period was shorter than SRG1 and vB_EfaS_HEf13 phages, and its burst size was smaller than theirs^8,21^. Large burst size may be an advantage for a phage to be used as an antibacterial agent in phage therapy since up to 100-fold of progeny can be produced in a short time^22^.

The results of analyzing the vB_EfaS-SRH2 genome indicated that it conforms to other members of *Siphoviridae* with ~ 50 kbp genome sizes such as vB_EfaS_PHB08 (Accession No. MK570225.1, Size: ~ 55 kbp), SANTOR1 (Accession No. KX284704, Size: ~ 38 kbp), and IME-EF1 (Accession No. KF192053.1, Size: ~ 57 kbp)^16,20^. Based on the BLASTN analysis, the nucleotide sequence of the vB_EfaS-SRH2 phage has high similarity to the genomes of vB_EfaS_IME196 (Identity: 92.17%, Query coverage: 82%, Accession No. KT932701.1, host: *E. faecalis*) and EfaCPT1 (Identity: 96.69%, Query coverage: 90%, Accession No. JX193904.1, host: *E. faecalis*) phages. According to the phylogenic relationship analysis of this phage with other phages using two genes coding for the capsid and tail family proteins, the capsid gene in the vB_EfaS-SRH2 phage has a close relationship with vB_EfaS-Max (Accession No. QAX97257.1). Furthermore, the tail family protein of this bacteriophage is closely associated with *Enterococcus* phage AUEF3 (Accession No. YP_009625860). During the biological evolutions, it is expected that viruses acquire anti-CRISPER codes to have priority to bacteria^23^. The vB_EfaS-SRH2 phage showed this valuable evolution with eight anti-CRISPERs in its genomic structure, which is a positive point for this phage. Moreover, there were no virulence factors including, antibiotic resistance, lysogenic, and toxin genes in the whole-genome sequence, so the phage could act well against the host and be qualified for phage therapy.

Comparing main proteins of the vB_EfaS-SRH2 phage indicated that helicase coding protein (ORF13) was 95% similar to the IME_EF4 phage. Moreover, DNA methylase coding protein (ORF17) was 100% similar to vB_EfaS_159 phage^24^. ORF34 and ORF35, which are related to the proteins coding the vB_EfaS-SRH2 phage tail, had high similarity to the counterparts in the vB_EfaS-IME196 phage. Besides, the ORF47 capsid gene had 99% similarity to the *Enterococcus* phage phiSHEF5^25^. Small and large subunits (ORF51 and ORF53) had 99% similarity to the *Enterococcus* phages IME-EF4 and EFAP-1^26^. ORF45 encoding DNA packaging protein had 98% similarity to *Enterococcus* phage phiSHEF4^25^. The longest ORF of vB_EfaS-SRH2 phage (ORF38) located in positive strand encoding the tail length tape-measure protein had 90% similarity to *Enterococcus* phage vB_EfaS_IME196. Therefore, altogether, the vB_EfaS-SRH2 phage can be considered as a new member of *Siphovirida*e.

The formation of biofilms on root canal walls^4^ and the high presence of *E. faecalis* in subgingival biofilms of periodontitis cases^27^ have already been reported. Biofilm matrix reduces the penetration of antibiotics. Thus, the cells can survive and increase resistance to antibiotics. Phages remove biofilms by their lysines and different types of depolymerases^28^. vB_EfaS-SRH2 reduced biofilm mass which is another advantage for the application of this phage for phage therapy.

## Conclusion

A specific bacteriophage with proper characteristics such as tolerating a wide range of pH, salt concentrations and temperatures, short latent period, large burst size and having eight anti-CRISPERs and lacking undesirable virulence, lysogenic, and antibiotic resistance genes was isolated against *E. faecalis*. Therefore, this phage can be considered as a proper candidate for phage therapy of odontogenic infections and resistant apical periodontitis caused by this bacterium.

## Methods

### *E. faecalis* isolation

This research was approved by the ethics committee of the University of Isfahan (IR.UI.REC.1399.058). According to the guidelines of the Declaration of Helsinki (Ethical Principles for Medical Research Involving Human Subjects), informed consents were obtained from all participants. Samples were taken from patients, including adults of both sexes aged 30–70 years old with periodontitis referred to the Periodontics Department of Dentistry Faculty, Isfahan University of Medical Sciences. For sampling, sterilized paper points (size 40) (Meta Biomed, South Korea) were inserted into pocket dept and put in tubes containing sterilized BHI broth (Merck, Germany). The samples were vortexed for 30 s, cultured on Mitis Salivarious Agar medium (Himedia, India), and incubated with 5% CO_2_ at 37 °C for 24 h under static conditions. *E. faecalis* strains were identified by individual colony morphology, Gram staining, and biochemical assays^29^. A confirmatory PCR test was performed for the final confirmation of identification. Bacterial DNA was extracted by the boiling method^30^. Then, PCR was performed using universal primers for 16S rRNA with a final product of ~ 370-bp^31^ (Supplementary Tables S2 and S3). The PCR products were sequenced using the Sanger method by Stabvida Company (Portugal) and identified by alignment using BLASTN at NCBI. MEGA7 software was used to draw phylogenetic trees. To draw the phylogenetic tree a set of sequences aligned using the ClustalW program and the maximum-likelihood method in MEGA 7 software with 1000 bootstrap values. Also, *Lactobacillus casei* (KF673501.1) was used as an out-group^32^.

### Antimicrobial susceptibility

Antibiotic resistance patterns of *E. faecalis* isolates were determined by disc diffusion method based on the CLSI protocol (Clinical and Laboratory Standards Institute)^33^ and using different antibiotics (ROSCO, Germany) (Supplementary Table S4). Müller-Hinton agar plates (USA, BioLife; MHA) were inoculated with the standard 0.5 McFarland suspension of the strains. The inhibition zone diameter around each disc was measured after 24 h of incubation at 37 °C.

### Phage isolation and purification

Wastewater samples were collected from different places in Isfahan. Each sample was separately centrifuged at 10,000×*g* for 10 min. Then, they were filtrated through a 0.45 μm membrane filter (Orang, Germany), and 30 mL of the filtrates were added to 30 mL 2 × BHI broth containing *E. faecalis* (ATCC 29212; IROST, Iran) and *E. faecalis* (Accession No.LC322250.1), which were at the early-exponential phase. These mixtures were incubated at 37 °C with shaking at 100 rpm for 24 h. Afterward, they were centrifuged at 12,000×*g* for 10 min at 4 °C, and the supernatants were filtered by a 0.45 μm membrane filter (Orang, Germany). Then, 20 μL of the filtrates were spotted on BHI agar (Merck, Germany) plates containing 0.7% agar and 1 × 10^8^ CFU/mL of *E. faecalis*, and incubated at 37 °C for 24 h. After observing plaques, to titer the virus, the supernatants of the prior steps were serially diluted (10^–1^ to 10^–10^) in SM buffer (100 mM NaCl; 8 mM MgSO_4_; 50 mM Tris–HCl; pH 7.5) and 10 μL of each serial dilutions were mixed with 0.1 mL of *E. faecalis* pre-logarithmic culture in 5 mL BHI agar containing 0.7% agar and poured on coagulated BHI containing 1.5% agar. After overnight incubation at 37 °C, one of the clear and small with smooth border plaques was collected by a sterile Pasteur pipette and homogenized in 50 μL of SM buffer. To purify the virus, then, 100 μL of each of the homogenous mixtures was added to 25 mL of BHI broth containing *E. faecalis* and incubated at 37 °C for 24 h. The two-layer agar method was repeated three times to ensure the isolation of purified phage^34^.

### Phage morphology

Phage morphology was assayed with a transmission electron microscope (Fei Philips TEM EM208S, Japan). One drop of the filtered phage with a high titer (approximately 1 × 10^16^ PFU/mL) was added to the surface of a copper grid (Ted Pella, USA) coated with carbon and kept at room temperature for 10 min. Then, dying was done with 2% (w/v) uranyl acetate (Sigma-Aldrich, USA). The stained grids were visualized at 100 kV^30^.

### Determination of the host range and EOP

Phage host range was examined using the spot test method on different strains of *E. faecalis* isolated from different periodontitis patients and 9 standard species (IROST, Iran) (Supplementary Table S5). For this, 10 μL of the isolated phage (1 × 10^9^ PFU/mL) was placed on BHI agar containing 100 μL (OD_600_ = 1) of each bacterial species, incubated at 37 °C for 24–48 h, and then investigated based on the inhibitory zone induced. Phage-sensitive species were evaluated by the efficiency of plating (EOP) calculations. EOP was calculated as (average PFU/mL on each isolates of *E. faecalis*/average PFU/mL on *E. faecalis* ATCC 29212) × 100. The EOP value obtained with the standard strain of *E. faecalis* was considered 100%^35^.

### Thermal and pH stability

One milliliter of the phage with 1 × 10^9^ PFU/mL concentration was incubated at different temperatures (− 20, 4, 22, 37, 40, 50, 60, and 70 °C) and titrated after 1, 6, and 12 h. For assessing pH stability, 100 μL of the phage (1 × 10^9^ PFU/mL) was added to 900 μL of SM buffer with different pH values (2–12), incubated at 37 °C, and examined after 1, 6, and 12 h. Then, the titers of the phage were determined by the two-layer agar method. The phages incubated at 37 °C and pH 7 were considered as the controls for the temperature and pH, respectively. All the experiments were performed in triplicate^30^.

### Determination of saline stress

The phage salt stability was determined in the presence of different concentrations of saline (1%, 5%, 10%, 20%, 30%, 40%, and 50%). For this, 100 μL of the phage with a titer of 1 × 10^9^ PFU/mL was added to 900 μL of each salt concentration. Then, serial dilutions of each saline concentration were prepared after 1, 6, and 12 h, and the titer of the phage was determined by the two-layer agar method. SM buffer was used as a control. During these experiments, the temperature and pH of the samples were kept at 25 °C and 7, respectively. This was repeated three times^30^.

### Absorption rate and one-step growth

To determine the absorption rate, 9 mL of overnight-cultured *E. faecalis* with OD_600_ = 0.7 was mixed with 1 mL of the phage (1 × 10^9^ PFU/mL) and incubated at 37 °C. Then, 100 μL of the mixture was collected every 2 min for 16 min. Each sample was centrifuged at 8000×*g* for 10 min. The titers of the non-absorbed phages in the supernatants were measured by the two-layer agar method. At time zero the host bacterium was blended with the phage. After adding of the phage to the *E. faecalis* (ATCC 29212; IROST, Iran) at time zero, the first sample was considered as 100% non-absorbed phages. The percent of the non-adsorbed phages at each time interval obtained from the ratio of PFU/mL of the non-absorbed phages to PFU/mL of non-absorbed phages at time zero^30,36^.

For one-step growth analysis, 0.5 mL of the bacterial culture (OD_600_ = 0.7) was centrifuged at 13,000×*g* for 10 min at 4 °C. The pellets were dissolved in 0.5 mL of fresh BHI broth medium and added to 0.5 mL of the phage (1 × 10^9^ PFU/mL). After 10 min, for adsorption of the phage to the bacterium, the suspension was centrifuged at 12,000×*g* for 10 min at 4 °C. The pellets were resuspended in 20 mL of fresh BHI broth and incubated at 37 °C. Then, 100 μL samples were taken every 5 min and immediately titrated by the two-layer agar method. Also, the second set of the samples were treated with 1% (vol/vol) chloroform before the phage titration to determine the eclipse period. Burst size was calculated as the ratio of the phage titer at plateau phase to the initial count of infected bacterial cells during the latent period^34,37^.

### Bacterial reduction assay

For this purpose, 900 μL of the bacterium at mid-log phase (OD_600_ = 0.7) was added in sterile tubes, and then, the phage suspensions with multiplicities of infection (MOIs) of 10, 1, 0.1, and 0.01 were added to each tube and incubated at 37 °C with shaking (110 rpm). The absorption rate was determined at OD 600 nm every 2 h for 24 h. A phage-free tube with MOI 0 was used as a control. Also, the phage titers (PFU/mL) and the survival of *E. faecalis* (CFU/mL) after phage infection were analyzed. To obtain the numbers of the viable cells and the phages per mL, samples were collected at 2 h intervals for 24 h and calculated based on counted colonies and counted plaques, respectively. As a control, *E. faecalis* was inoculated to culture medium without the phage. This was repeated three times^34,36^.

### Phage genomic DNA extraction

The genomic DNA of the phage was extracted by precipitation with acetone and extracted with potassium iodide according to Soleimani-Delfan et al. method^38^. For this, *E. faecalis* was first propagated in BHI broth (OD_600_ = 0.7), shaking at 110 rpm for 6 h at 37 °C. Then, 10 μL/mL DNAse I (Takara Bio, Japan) and 10 μL/mL RNase I (Takara Bio, Japan) were added to phage suspension and incubated at 37 °C for 30 min. To deactivate the ribonuclease and deoxyribonucleic enzymes, it was further incubated at 75 °C for 20 min. Next, 16 mL of pure acetone (pH 5.5) was added to precipitate 4 mL of the filtrated phage (Orang, Germany). It was then shaken vigorously and centrifuged at 10,000×*g* for 2 min to precipitate phage particles. The supernatant was discarded, and the pellet was kept at room temperature for a few minutes. The potassium iodide method was used for the phage DNA extraction by the addition of an equal volume of 3 M potassium iodide (Sigma, USA) (pH 6.5) and shaking at 4 °C for 1 min. It was then transferred to a silica-based spin column and centrifuged at 9000×*g* for a short time. The column was washed with washing buffer I (10 mM NaCl, 1 mM Tris–HCl, pH 7.5 in 80% ethanol), spun at 10,000×*g* for 1 min, and then washed for another 1 min at 10,000×*g* with washing buffer II (95% ethanol). Finally, the washed DNA was transferred to DNA collection microtubes and after adding 50 μL of sterile water, centrifuged at 12,500×*g* for 2 min. To evaluate the accuracy of the performance, it was loaded on 0.7% agarose gel. The extracted DNA was stored at − 20 °C.

### Genome sequencing and analyses

Whole-genome DNA was sequenced using the illumine Hiseq (2005) method by Novogene Company (China). The obtained sequence was assembled using Abyss paralleled Version 2.0.2 (http://www.bcgsc.ca/platform/bioinfo/Software/abyss)^39^. Two online software of PHASTER (PHAge Search Tool—Enhanced Release) (http://phast.wishartlab.com/)^40^ and GenMarkS version 3.25 (http://exon.gatech.edu/GeneMark/heuristic_gmhmmp.cgi)^41^ were used to predict ORFs. Then, the translation of each ORF to protein sequence was examined by Expasy translate tool (http://web.Expasy.org/Translate/)^42^. The putative function of each translated product was analyzed by BLASTP at NCBI (http://blast.ncbi.nlm.nih.gov/)^43^. GtRNAdb (http://gtrnadb.ucsc.edu)^44^ and tRNAscan-SE (http://lowelab.ucsc.edu/tRNAscan-SE)^45^ software were used to predict tRNA encoding sequences. ARNold (http://rna.igmors.upsud.fr/toolbox/Arnold/)^46^ was used to obtain Rho-dependent termination sequences. Anti-CRISPER proteins were detected by Anti-CRISPERdp (http://bcb.unl.edu/AcrFinder)^47^. DNA Plotter software was used to draw a physical map of the genome^48^. The ARDB database (http://ardb.cbcb.umd.edu)^49^ and ResFinder 4.0 (https://cge.cbs.dtu.dk/services/ResFinder-4.0/)^50^ were used to identify antibiotic resistance genes and virulence factors. Using Viptree^51^, the whole genome of the vB_EfaS-SRH2 phage sequence was compared to other reported phages of *E. faecalis* at NCBI. Phylogenetic analysis was conducted based on amino acid sequences of capsid protein (ORF 47) and tail family protein (ORF 37) of the phage compared to other *Siphoviridae* phages using MEGA 7 software^32^. Respectively, the capsid and tail family proteins *of Lactococcus garvieae* phage WP-2 (Accession No.: AHZ10895.1) and *Salmonella* phage vB_StyS-sam (Accession No.: BBO65982.1) were used as out-groups. The whole-genome sequence of the *E. faecalis* bacteriophage was deposited in GenBank (Accession No.: LC623721.1).

### Biofilm removal

*E. faecalis* was cultured overnight in 10 mL of BHI broth and incubated at 37 °C for 24 h (OD_600_ = 0.7). Then, it was shaken (220 rpm) at room temperature for 10–12 h. The bacterium (100 μL) was added to the wells of polystyrene microtitre plates (ExtraGene, Taiwan) and incubated at 37 °C for 24 h for the formation of biofilm. Then, 10 μL of the phage suspension (1 × 10^9^ PFU/mL) was added to each well and incubated at 37 °C for 24 h. After this, the contents were discarded, and the wells were washed with deionized water and dried in ambient air. The biomass was stained with 200 μL of crystal violet 0.1% for 5 min, washed again with deionized water, and dried in the ambient air. Finally, 200 μL of 70% ethanol was added to the wells; the optical density (OD) was determined at 600 nm wavelength, and read by a Multi-Mode Microplate Reader (BioTek, USA). Each assay was performed at least three times. Phage untreated biofilm and culture medium without bacteria were used as controls^52^.

### Statistical analyses

Data were analyzed by GraphPad Prism version 8.0 software (GraphPad Software Inc., USA) using one-way analysis of variance (ANOVA).

## Supplementary Information

Supplementary Information.

## Data Availability

The complete genome sequence of *Enterococcus* phage vB_EfaS-SRH2 and 16S rRNA sequences of *E. faecalis* are available in DNA DataBank of Japan (DDBJ)/European Nucleotide Archive (ENA)/GenBank under the names (accession numbers) vB_EfaS-SRH2 (LC623721.1) https://www.ncbi.nlm.nih.gov/nuccore/?term=vB_EfaS-SRH2, *Enterococcus faecalis* E.F (Isfahan) (LC322250.1) https://www.ncbi.nlm.nih.gov/nuccore/LC322250.1, *Enterococcus faecalis* E.F2 (Isfahan) (LC414618.1) https://www.ncbi.nlm.nih.gov/nuccore/LC414618.1, *Enterococcus faecalis* E.F3 (Isfahan) (LC414619.1) https://www.ncbi.nlm.nih.gov/nuccore/LC414619, *Enterococcus faecalis* E.F4 (Isfahan) (LC414620.1) https://www.ncbi.nlm.nih.gov/nuccore/LC414620 and *Enterococcus faecalis* E.F5 (Isfahan) (LC414621.1) https://www.ncbi.nlm.nih.gov/nuccore/LC414621.
