# Supplementary material for: Isolation, characterization and complete genome analysis of a novel bacteriophage vB_EfaS-SRH2 against Enterococcus faecalis isolated from periodontitis patients
Source: Sci Rep. 2022 Aug 2;12:13268. doi: 10.1038/s41598-022-16939-0 (PMC9346004; doi:10.1038/s41598-022-16939-0)
Supplement: Supplementary file 1 — Supplementary Information. [file 41598_2022_16939_MOESM1_ESM.pdf]

Title: Isolation, characterization and complete genome analysis of a novel bacteriophage vB_EfaS-SRH2 against *Enterococcus faecalis* isolated from periodontitis patients

Setareh Pazhouhnia[a], Majid Bouzari[b,*], Farahnaz Arbabzadeh-Zavareh [c]

[a] Setareh Pazhouhnia, PhD student, Department of Cell and Molecular Biology & Microbiology, Faculty of Biological Science and Technology, University of Isfahan. Isfahan, Iran. Email: s.pazhouhnia@gmail.com

[b] Majid Bouzari, PhD, Professor of Virology, Department of Cell and Molecular Biology & Microbiology, Faculty of Biological Science and Technology, University of Isfahan. Isfahan, Iran. Email: bouzari@sci.ui.ac.ir or mbouzari@yahoo.com

[c] Farahnaz Arbabzadeh-Zavareh, DDS, MSc, Associate Professor of Restorative Dentistry, Department of Operative Dentistry and Torabinejad Dental Research Center, School of Dentistry, Isfahan University of Medical Sciences, Isfahan, Iran. Email: arbabzadeh@dnt.mui.ac.ir

***Corresponding author**: Professor Majid Bouzari, Department of Cell and Molecular Biology & Microbiology, Faculty of Biological Science and Technology, University of Isfahan., Hezar-Jereeb Street, 81746-73441, Isfahan, Iran. Email: bouzari@sci.ui.ac.ir or mbouzari@yahoo.com.

Supplementary figures and tables:

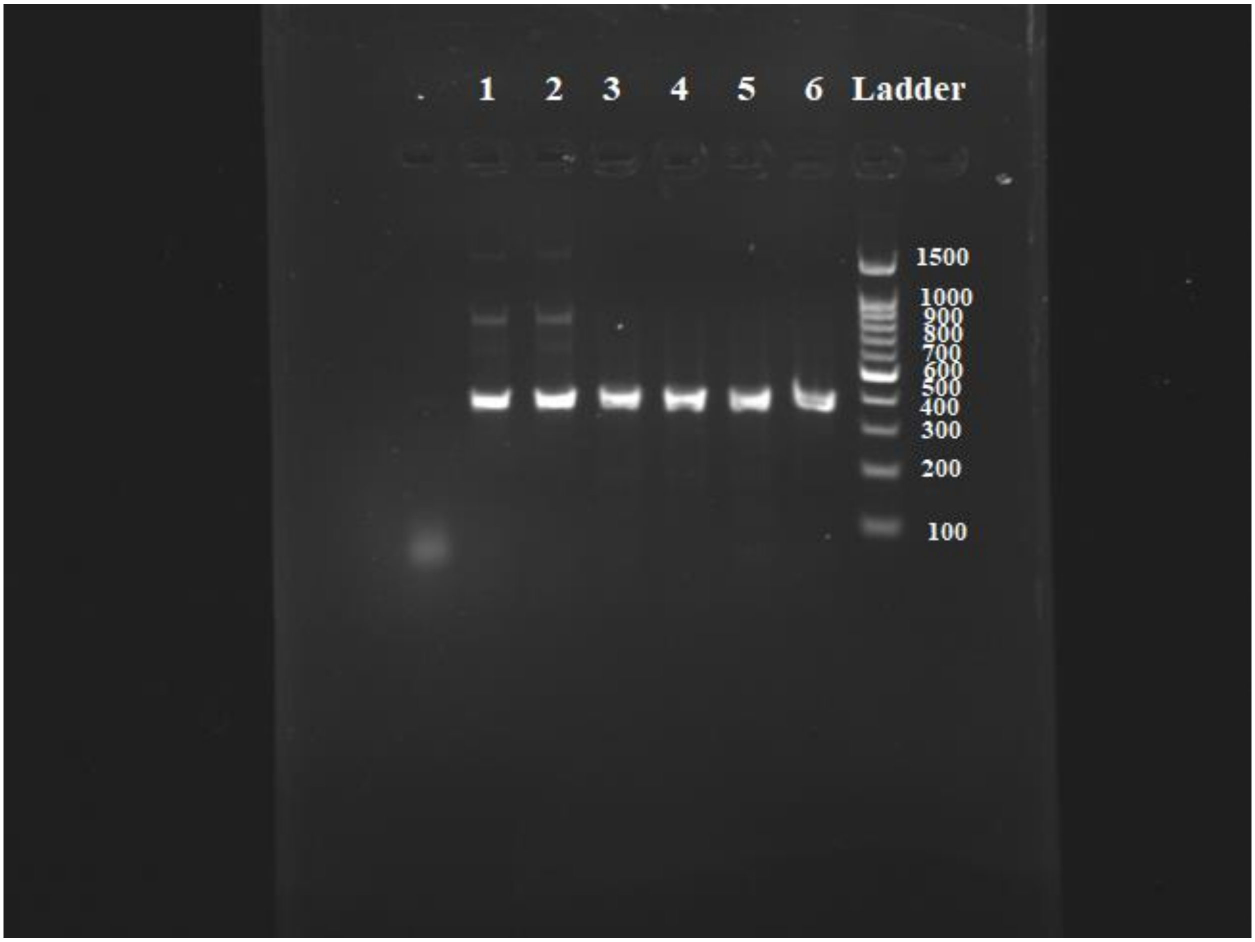

Supplementary Fig. 1. Gel electrophoresis of the PCR products of 16S rRNA of the isolated *E. faecalis* species. 1: Positive control, 2: LC322250.1, 3: LC414618.1, 4: LC414619.1, 5: LC414620.1, and 6: LC414621.1.

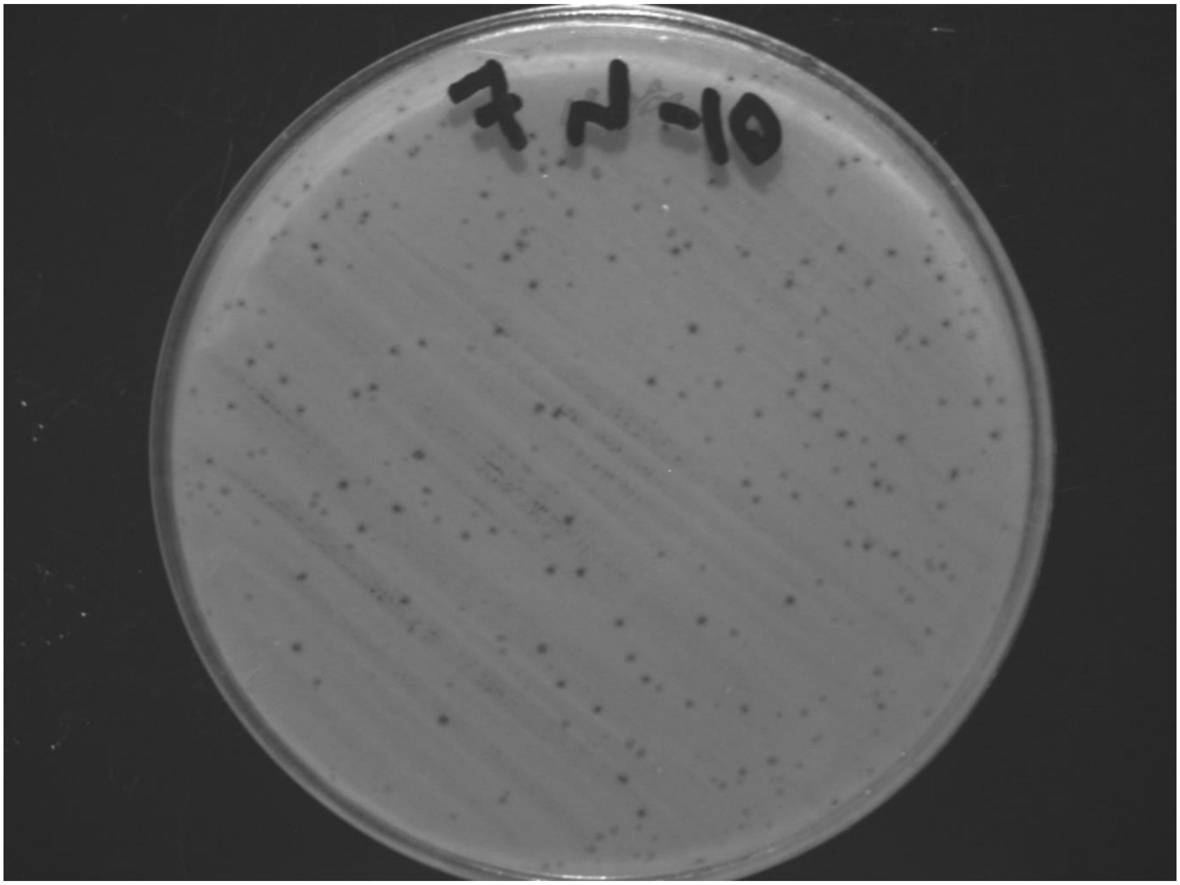

Supplementary Fig. 2. Plaques formed in two-layer agar plates by the isolated phage (vB_EfaS-SRH2).

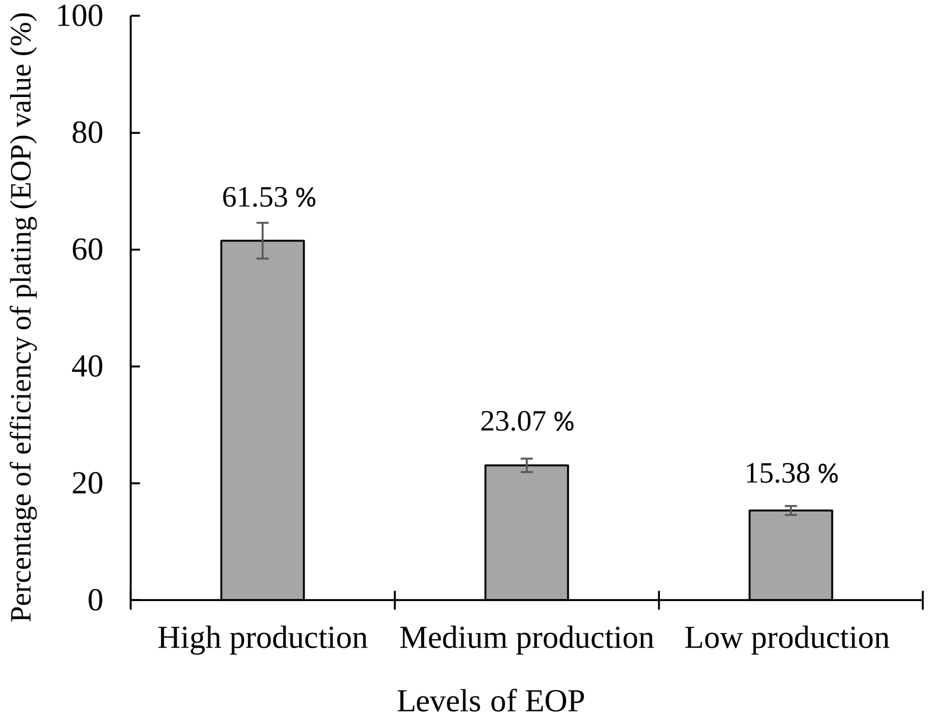

Supplementary Fig. 3. The efficiency of plating (EOP) obtained for different *E. faecalis* strains. EOP values are the mean of three independent assays. Error bars represent the standard deviation. High production (≥ 0.5), medium production (0.2 - 0.5) and low production (0.2 - 0.001).

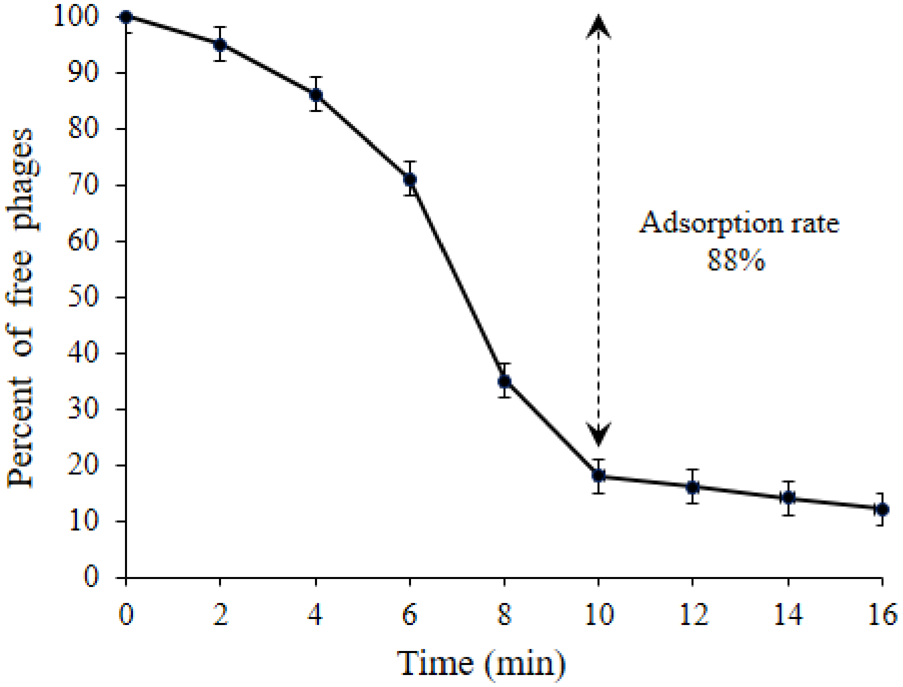

Supplementary Fig. 4. Adsorption rate of the vB_EfaS-SRH2 phage on the *E. faecalis* cells.

The data represent the mean from triplicate experiments. Error bars represent the standard deviation.

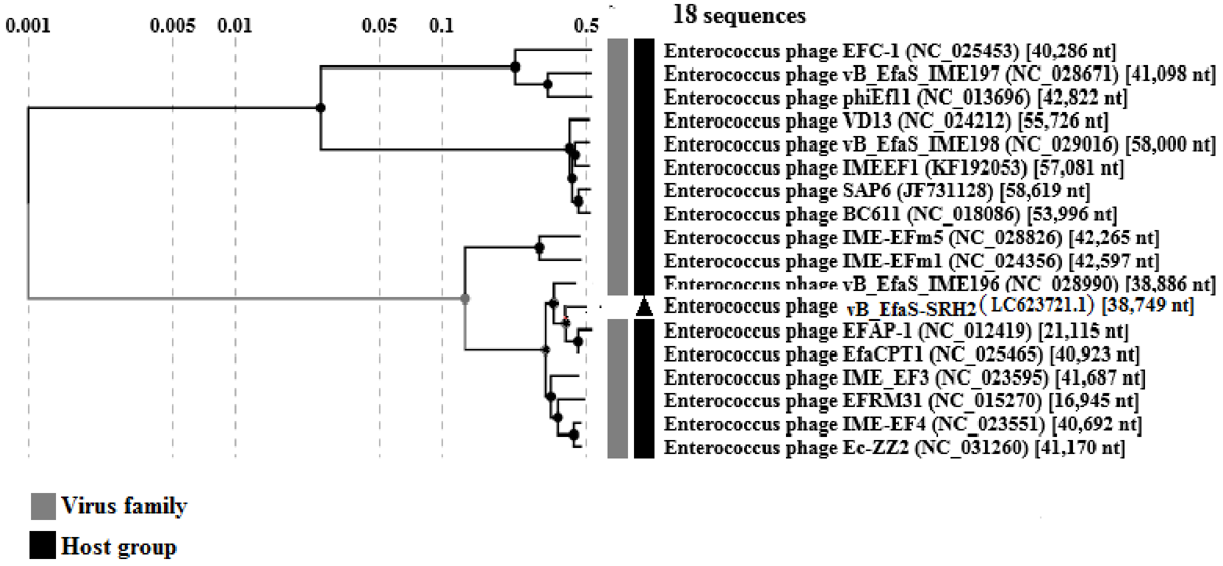

Supplementary Fig. 5. Map of the bacteriophage vB_EfaS-SRH2 genome created using the VIP tree software. Showing relationship between vB_EfaS-SRH2 phage and other Enterococcus phages of the *Siphoviridae* family

▲: sequence of the vB_EfaS-SRH2 phage detected in this study.

Supplementary Table 1. Phage vB_EfaS-SRH2 gene annotations.

ORF	Position (nt)		Strand	Codon		Size (aa)[1]	Predictive Function	Closest hit (accession number)	Identity	E value	Mw[2] (Da)	pI[3]	Accession Number
	From	To		Start	Stop								
1	90	206	+	ATG	TAG	38	Hypothetical protein	Hypothetical protein AVT93_gp50 [Enterococcus phage vB_EfaS_IME196] YP_009216637.1	84.62%	8e-07	4404.21	8.16	**BCU01236.1**
2	221	373	+	ATG	TAA	50	Hypothetical protein	Hypothetical protein BI091_gp48 [Enterococcus phage SANTOR1] YP_009284759.1	98.00%	1e-26	6009.09	9.87	**BCU01237.1**
3	373	594	+	ATG	TAG	73	Hypothetical protein	Hypothetical protein BJD48_gp47 [Enterococcus phage Ec-ZZ2] YP_009303746.1	93.15%	2e-40	8577.87	4.28	**BCU01238.1**
4	809	1033	+	ATG	TGA	74	Hypothetical protein	Hypothetical protein FDI48_gp53 [Enterococcus phage phiSHEF2] YP_009613333.1	85.33%	1e-35	8619.61	4.07	**BCU01239.1**
5	1030	1227	+	ATG	TAA	65	Hypothetical protein	Hypothetical protein [Enterococcus phage vB_EfaS_Ef5.3] QBZ70277.1	95.38%	2e-25	6936.73	8.16	**BCU01240.1**
6	1322	2902	+	ATG	TAA	526	DNA primase/helicase, phage-associated	DNA primase/helicase, phage-associated [Enterococcus phage Q69] CAD0300158.1	98.86%	0.0	60504.31	5.62	**BCU01241.1**
7	2992	3180	+	ATG	TAA	62	Hypothetical protein	Hypothetical protein CH14_gp18 [Enterococcus phage IME-EF4] YP_009004329.1	98.39%	6e-34	7410.50	5.35	**BCU01242.1**
8	3254	3715	+	ATG	TAA	153	Hypothetical protein	Hypothetical protein BJD48_gp54 [Enterococcus phage Ec-ZZ2] YP_009303753.1	98.69%	6e-107	17746.44	9.75	**BCU01243.1**
9	3848	4132	+	ATG	TAA	94	Hypothetical protein	hypothetical protein BJD48_gp55 [Enterococcus phage Ec-ZZ2] YP_009303754.1	98.94%	2e-55	10436.93	4.93	**BCU01244.1**
10	4134	4295	+	ATG	TAA	53	Hypothetical protein	Hypothetical protein BJD48_gp56 [Enterococcus phage Ec-ZZ2] YP_009303755.1	98.11%	7e-27	5762.71	3.83	**BCU01245.1**
11	4297	4509	+	ATG	TAA	70	Hypothetical protein	Hypothetical protein AVT93_gp41 [Enterococcus phage vB_EfaS_IME196] YP_009216628.1	98.57%	2e-40	8018.06	3.98	**BCU01246.1**
12	4509	4895	+	ATG	TAA	128	Endonuclease	Endonuclease [Enterococcus phage Sigurd] QYW02436.1	100%	6e-69	14721.59	4.28	**BCU01247.1**

13	4810	6183	+	ATG	TGA	457	DNA helicase	Helicase [Enterococcus phage IME-EF4] YP_009004335.1	95.82%	0.0	53017.77	9.38	**BCU01248.1**
14	6180	6356	+	ATG	TAG	58	Hypothetical protein	Hypothetical protein EfaCPT1_gp37 [Enterococcus phage EfaCPT1] YP_009103854.1	98.28%	1e-31	6845.98	9.22	**BCU01249.1**
15	6419	6616	+	ATG	TAA	65	Hypothetical protein	Hypothetical protein TV217_37 [Enterococcus phage vB_EFaS_TV217] QMS41987.1	96.92%	1e-39	7850.03	4.82	**BCU01250.1**
16	6629	7372	+-	ATG	TAA	247	Hypothetical protein	|Hypothetical protein [Enterococcus phage vB_EfaS_Ef5.2] QBZ69975.1	96.36%	3e-175	27967.61	5.65	**BCU01251.1**
17	7323	8111	+	ATG	TAA	262	DNA methylase	DNA methylase [Enterococcus phage 9184] QOI68860.1	92.06%	2e-108	30456.73	8.90	**BCU01252.1**
18	8059	8310	+	ATG	TAA	83	Hypothetical protein	Hypothetical protein heks_48 [Enterococcus phage heks] QIQ66151.1|	98.53%	2e-42	9719.50	9.41	**BCU01253.1**
19	8101	8310	+	ATG	TAA	68	XRE family transcriptional regulator	XRE family transcriptional regulator [Enterococcus phage MSF2] QDF15331.1	98.41%	4e-38	80.35	8.79	**BCU01254.1**
20	8467	8913	+	ATG	TAA	148	Hypothetical protein	Hypothetical protein BI091_gp31 [Enterococcus phage SANTOR1] YP_009284742.1	93.22%	2e-30	17277.77	5.97	**BCU01255.1**
21	8915	9121	+	ATG	TGA	68	Hypothetical protein	Hypothetical protein FDI49_gp25 [Enterococcus phage phiSHEF4] YP_009613373.1	98.53%	4e-41	7934.14	4.71	**BCU01256.1**
22	9118	9597	+	ATG	TAG	159	putative HNH homing endonuclease-like protein	Putative HNH homing endonuclease-like protein [Enterococcus phage Ec-ZZ2] YP_009303708.1	99.37%	2e-113	18658.03	7.77	**BCU01257.1**
23	9608	10384	+	ATG	TGA	258	putative beta-lactamase superfamily domain protein	Putative metallo-beta-lactamase domain protein [Enterococcus phage EfaCPT1] YP_009103846.1	96.12%	0.0	30043.87	5.67	**BCU01258.1**
24	10360	10548	+	ATG	TGA	62	Hypothetical protein	Hypothetical protein CH14_gp34 [Enterococcus phage IME-EF4] YP_009004345.1	98.39%	7e-36	7481.70	5.33	**BCU01259.1**
25	11357	11608	+	ATG	TAG	83	Hypothetical protein	Hypothetical protein BI091_gp25 [Enterococcus phage SANTOR1] YP_009284736.1	99.10%	7e-161	9669.04	4.52	**BCU01260.1**
26	11630	12391	+	ATG	TAA	253	Hypothetical protein	Hypothetical protein EfaCPT1_gp24 [Enterococcus phage EfaCPT1] YP_009103841.1	99.21%	0.0	28646.47	4.74	**BCU01261.1**
27	11684	12391	+	ATG	TAA	235	Hypothetical protein	Hypothetical protein [Enterococcus phage vB_EfaS_Ef5.3] QBZ70254.1	99.57%	3e-169	26430.83	4.66	**BCU01262.1**

28	12456	12686	+	ATG	TAA	74	Hypothetical protein	Hypothetical protein FDI50_gp46 [Enterococcus phage phiSHEF5] YP_009613457.1	98.68%	1e-46	8542.97	9.42	**BCU01263.1**
29	12722	15013	+	ATG	TAA	763	DNA polymerase B region	DNA polymerase B region [Enterococcus phage vB_EfaS_159] CAD0310631.1	98.69%	0.0	87627.67	5.46	**BCU01264.1**
30	15076	15675	+	ATG	TGA	199	DNA modification protein	DNA modification [Enterococcus phage vB_EfaS_Ef5.2] QBZ69964.1	97.99%	3e-141	22956.51	9.20	**BCU01265.1**
31	15672	15908	+	ATG	TAG	78	Gutaredoxin-like protein	Glutaredoxin family protein [Enterococcus phage AUEF3] YP_009625866.1	100.00%	2e-49	8950.39	5.05	**BCU01266.1**
32	16981	15998	-	ATG	TAA	327	lysin, N-acetylmuramoyl-L-alanine amidase	Lysin, N-acetylmuramoyl-L-alanine amidase family protein [Enterococcus phage AUEF3] YP_009625865.1	99.69%	0.0	36448.91	5.92	**BCU01267.1**
33	17217	16984	-	ATG	TAA	77	Holin	Holin [Enterococcus phage vB_EfaS_AL3] YP_009624747.1	100.00%	5e-45	8461.08	8.06	**BCU01268.1**
34	17477	17232	-	ATG	TAA	81	Tail fiber protein	Tail fiber protein [Enterococcus phage vB_EfaS_IME196] YP_009216606.1	100.00%	7e-50	9096.47	5.23	**BCU01269.1**
35	17987	17655	-	ATG	TAG	110	Hypothetical protein	Hypothetical protein AVT93_gp18 [Enterococcus phage vB_EfaS_IME196] YP_009216605.1	93.68%	9e-61	12676.28	4.58	**BCU01270.1**
36	20014	17957	-	ATG	TAG	685	Endopeptidase tail	Endopeptidase tail [Enterococcus phage phiSHEF2] YP_009613297.1	93.58%	0.0	77105.29	5.05	**BCU01271.1**
37	22169	20085	-	ATG	TAA	694	Tail family protein	Tail family protein [Enterococcus phage AUEF3] YP_009625860.1	90.35%	0.0	79931.84	5.00	**BCU01272.1**
38	26612	22242	-	ATG	TAA	1456	Tail length tape-measure protein	Tail length tape-measure protein [Enterococcus phage vB_EfaS_IME196] YP_009216602.1	90.66%	0.0	156368.99	9.46	**BCU01273.1**
39	27180	26869	-	ATG	TAG	103	Putative tail tape measure chaperone protein	Putative tail tape measure chaperone protein [Enterococcus phage EFA1] QOI58196.1	100.00%	2e-67	11621.30	4.46	**BCU01274.1**
40	27584	27375	-	ATG	TAG	69	Major tail protein	Phi13 family major tail protein [Enterococcus phage AUEF3] YP_009625924.1	100.00%	1e-40	7497.30	4.89	**BCU01275.1**
41	28382	28017	-	ATG	TAA	121	Head-tail joining protein	Head-tail joining protein [Enterococcus phage vB_EfaS_LM99] AXC33940.1	100.00%	1e-84	14196.07	4.78	**BCU01276.1**
42	28786	28379	-	ATG	TGA	135	Hypothetical protein	hypothetical protein FDI50_gp58 [Enterococcus phage phiSHEF5] YP_009613469.1	100.00%	1e-95	15333.40	9.02	**BCU01277.1**

43	29118	28783	-	ATG	TGA	111	Head-tail adaptor protein	Head-tail adaptor protein [Enterococcus phage FX417] QOI67934.1	97.30%	4e-75	13384.23	7.76	**BCU01279.1**
44	29121	28783	-	ATG	TGA	112	Head-tail joining protein	Head-tail joining protein [Enterococcus phage phiSHEF2] YP_009613290.1	100.00%	9e-77	13515.42	7.76	**BCU01278.1**
45	29392	29090	-	ATG	TGA	100	DNA packaging protein	DNA packaging protein [Enterococcus phage PMBT2] YP_009622333.1	98.98%	4e-64	11534.23	4.47	**BCU01280.1**
46	29630	29430	-	ATG	TAA	66	Putative major tail protein	Major tail protein [Enterococcus phage IME-EF4] YP_009004365.1	89.39%	4e-33	7023.97	7.94	**BCU01281.1**
47	31009	29756	-	ATG	TAG	417	Capsid family protein	Capsid family protein [Enterococcus phage phiSHEF5] YP_009613473.1	99.04%	0.0	46922.81	7.73	**BCU01282.1**
48	31667	31080	-	ATG	TAA	195	Head maturation protease	Head maturation protease [Enterococcus phage vB_EfaS_IME196] YP_009216592.1	98.40%	3e-130	22121.28	5.25	**BCU01283.1**
49	32781	31630	-	ATG	TGA	383	Portal protein	Putative portal protein [Enterococcus phage EfaCPT1] YP_009103820.1	100.00%	0.0	42903.42	5.02	**BCU01284.1**
50	32786	32974	-	ATG	TAA	62	Hypothetical protein	Hypothetical protein [Enterococcus phage vB_EfaS_Ef5.4] QBZ69813.1	98.39%	1e-32	6676.14	4.78	**BCU01285.1**
51	34744	33020	-	ATG	TAA	574	Terminase large subunit	Terminase large subunit [Enterococcus phage PMBT2] YP_009622327.1	100.00%	0.0	65790.22	5.32	**BCU01286.1**
52	35110	34934	-	ATG	TAG	58	Hypothetical protein	Hypothetical protein [Enterococcus phage vB_EfaS_Ef5.4] QBZ69811.1	96.55%	1e-31	6551.70	10.29	**BCU01287.1**
53	35702	35229	-	ATG	TAG	157	Terminase small subunit	Terminase small subunit [Enterococcus phage EFAP-1] YP_002727893.1	99.36%	4e-111	18123.47	5.21	**BCU01288.1**
54	36040	35705	-	ATG	TAA	111	Endodeoxyribonuclease	Putative endodeoxyribonuclease [Enterococcus phage phiNASRA1] QEL53371.1	98.20%	2e-72	12835.42	9.70	**BCU01289.1**
55	36192	36040	-	ATG	TAA	50	Hypothetical protein	Hypothetical protein CF83_gp02 [Enterococcus phage IME_EF3] YP_009008901.1	100.00%	4e-28	5905.73	9.82	**BCU01290.1**
56	36421	36119	-	ATG	TAA	100	Hypothetical protein	Hypothetical protein EfaCPT1_gp67 [Enterococcus phage EfaCPT1] YP_009103884.1	97.75%	5e-55	11709	10.03	**BCU01291.1**
57	36353	36231	-	ATG	TAG	123	Hypothetical protein	Hypothetical protein [Enterococcus phage vB_EfaS_Ef5.4] QBZ69808.1	97.50%	1e-19	5109.05	9.43	**BCU01292.1**

58	36955	36584	-	ATG	TGA	123	HNH endonuclease	HNH endonuclease [Enterococcus phage EFAP-1] YP_002727895.1	98.37%	2e-86	14699.62	9.17	**BCU01293.1**
59	37173	36955	-	ATG	TAA	72	Hypothetical protein	Hypothetical protein BI091_gp59 [Enterococcus phage SANTOR1] YP_009284770.1	91.67%	7e-38	8209.23	4.91	**BCU01294.1**
60	37418	37215	-	ATG	TAA	67	Hypothetical protein	Hypothetical protein EfaCPT1_gp64 [Enterococcus phage EfaCPT1]; YP_009103881.1	99.45%	3e-129	7584.27	9.52	**BCU01295.1**
61	38608	38048	+	ATG	TAA	186	Hypothetical protein	Hypothetical protein [Enterococcus phage vB_EfaS_Ef5.2] QBZ70003.1	91.01%	2e-100	21982.14	8.82	**BCU01296.1**
62	38732	38616	+	ATG	TGA	38	Hypothetical protein	Hypothetical protein [Enterococcus phage EFA1] QOI58233.1	80.00%	4e-08	64418.28	9.30	**BCU01297.1**

[1]amino acids, [2] Molecular weight, [3] pH isoelectric

Supplementary Table 2. PCR reagents and program used in this study.

Reagent	Volume
DNA template	2 µL
dNTPs (40 µM)	0.5 µL
10x PCR buffer (75 mM Tris-HCl, pH 9.0, 2 mM of $MgCl_2$, 50 mM of KCl, 20 mM of $(NH4)_2SO_4$)	2.5 µL
Ampli *Taq* DNA polymerase (1U/ µL) (Fermentas, USA)	0.5 µL
Forward primer (RW01) (20 pmol)	0.5 µL
Reverse primer (DG74) (20 pmol)	0.5 µL
Sterilized Water	18.5 µL
Total volume	25 µL

Supplementary Table 3. PCR program used in this study.

Step	Temperature	Time	Number of Cycles
**Initial denaturation**	95 °C	5 min	1
**Denaturation**	94 °C	45 sec	
**Annealing**	54 °C	30 sec	30
**Extension**	72 °C	45 sec	
**Final extension**	72 °C	5 min	1

Supplementary Table 4. Antibiotics used to evaluate antimicrobial susceptibility.

**Antibiotic**	**Abbreviation**	**Concentration**
Penicillin	PEN	6 µg
Amoxicillin	AMX	25 µg
Ampicillin	AMP	10 µg
Amoxicillin/clavulanic acid	AMC	20/10 µg
Amikacin	AMK	30 µg
Gentamicin	GEN	10 µg
Kanamycin	KAN	500 µg
Streptomycin	STP	10 µg
Erythromycin	ERY	15 µg
Nalidixic acid	NAL	30 µg
Ciprofloxacin	CIP	5 µg
Vancomycin	VAN	30 µg
Chloramphenicol	CHL	30 µg
Tetracycline	TET	30 µg
Clindamycin	CLI	2 µg
Oxacillin	OXA	1 µg

Supplementary Table 5. Bacterial species used to assess host range.

**Bacterial species**	**ATCC and accession number**	**Clear zone**
E. faecalis	ATCC 29212	+
Pseudomonas aeruginosa	ATCC 27853	-
Escherichia coli	ATCC 25922	-
Salmonella typhimurium	ATCC 14028	-
Shigella sonnei	ATCC 12022	-
Staphylococcus aureus	ATCC 35933	-
Streptococcus mutans	ATCC 35668	-
Streptococcus salivarius	ATCC 9222	-
Bacillus cereus	ATCC 14579	-
E. faecalis EF (Isfahan)	LC322250.1	+
E. faecalis EF2 (Isfahan)	LC414618.1	+
E. faecalis EF3 (Isfahan)	LC414619.1	+
E. faecalis EF4 (Isfahan)	LC414620.1	+
E. faecalis EF5 (Isfahan)	LC414621.1	+

+: Presence of clear zone

-: Absence of clear zone
